# Mind the gap: knowledge, attitudes and perceptions on antimicrobial resistance, antimicrobial stewardship and infection prevention and control in long-term care facilities for people with disabilities in the Netherlands

**DOI:** 10.1186/s13756-024-01415-3

**Published:** 2024-06-05

**Authors:** S. Hidad, S. C. de Greeff, A. Haenen, F. de Haan, G. L. Leusink, A. Timen

**Affiliations:** 1 Center for Infectious Disease Control, National Institute for Public Health and the Environment, Bilthoven, The Netherlands; 2 Department of Primary and Community Care, Radboud University Medical Center, Nijmegen, the Netherlands

**Keywords:** Long-term care, Intellectual disability, Infection prevention and control, Antimicrobial resistance, Healthcare professionals, Antimicrobial stewardship

## Abstract

**Background:**

Antimicrobial resistance (AMR) has become one of the major public health threats worldwide, emphasizing the necessity of preventing the development and transmission of drug resistant microorganisms. This is particularly important for people with vulnerable health conditions, such as people with intellectual disabilities (ID) and long-term care residents. This study aimed to assess the current status of AMR, antimicrobial stewardship (AMS) and infection prevention and control (IPC) in Dutch long-term care facilities for people with intellectual disabilities (ID-LTCFs).

**Methods:**

A web-based cross-sectional survey distributed between July and November 2023, targeting (both nonmedically and medically trained) healthcare professionals working in ID-LTCFs in The Netherlands, to study knowledge, attitudes and perceptions regarding AMR, AMS and IPC.

**Results:**

In total, 109 participants working in 37 long-term care organizations for people with intellectual disabilities throughout the Netherlands completed the questionnaire. The knowledge levels of AMR and IPC among nonmedically trained professionals (e.g., social care professionals) were lower than those among medically trained professionals (*p = 0.026*). In particular regarding the perceived protective value of glove use, insufficient knowledge levels were found. Furthermore, there was a lack of easy-read resources and useful information regarding IPC and AMR, for both healthcare professionals as well as people with disabilities. The majority of the participants (> 90%) reported that AMR and IPC need more attention within the disability care sector, but paradoxically, only 38.5% mentioned that they would like to receive additional information and training about IPC, and 72.5% would like to receive additional information and training about AMR.

**Conclusion:**

Although the importance of AMR and IPC is acknowledged by professionals working in ID-LTCFs, there is room for improvement in regards to appropriate glove use and setting-specific IPC and hygiene policies. As nonmedically trained professionals comprise most of the workforce within ID-LTCFs, it is also important to evaluate their needs. This can have a substantial impact on developing and implementing AMR, AMS and/or IPC guidelines and policies in ID-LTCFs.

**Supplementary Information:**

The online version contains supplementary material available at 10.1186/s13756-024-01415-3.

## Background

Antimicrobial resistance (AMR) has become a major global health threat according to the World Health Organization (WHO) [1, 2]. Due to AMR, infections have become untreatable or more difficult to treat using antimicrobial agents [3]. Therefore, adequate infection prevention and control (IPC) practices and antibiotic stewardship programs (AMS) are required to improve or maintain individual healthcare outcomes, and to reduce the spread of resistant pathogens [1, 4–6]. This is particularly important for institutionalized individuals (e.g., those who live closely together and share facilities) and vulnerable people with (multiple) underlying diseases (e.g., chronic diseases and comorbidities).

People with intellectual disabilities (ID) are a poorly recognized risk group for health-related problems [7–9]. Previous research has demonstrated that people with ID encounter 2.5 times more health problems than people without ID and that chronic illnesses and comorbidities are more prevalent among this group [10]. Furthermore, people with ID were disproportionately affected during the COVID-19 pandemic in terms of mortality, further highlighting the vulnerability of this population [11]. Given their vulnerable health conditions and unacknowledged health needs, this group is hypothetically at greater risk for infections and AMR.

The prevalence of ID among countries worldwide ranges between 1% and 3% [7, 12, 13]. People with ID are characterized by notable impairments originating from childhood in both intellectual functioning and adaptive behavior [14]. Approximately 1.45% of the Dutch population, accounting for approximately 187,000 individuals, is estimated to have an ID [12]. In addition, 1.1 million people exhibit ID-related symptoms (IQ between 50 and 85 and/or difficulties with adaptive functioning) [12, 15]. Among individuals with an ID diagnosis, 63,000 people receive care and live within a long-term care facility under the Long-Term Care Act (WLZ act) [16, 17]. Medical care within long-term care facilities is provided by an intellectual disability specialist (ID-physician) and a general practitioner (GP) [18, 19]. Compared to other long-term care settings such as nursing homes for older adults, which have been previously described in literature, people with ID in long-term care facilities (ID-LTCFs) often live lifelong in the care facilities, whereas the average length of stay in long-term care for older adults in the Netherlands is two to four years [20]. People with intellectual disabilities residing in ID-LTCFs typically live in institutional settings but actively participate in society as much as possible [21]. However, due to the shared living conditions, multiple contact moments with healthcare professionals, and low basic hygiene and infection control compliance by people with ID due to limited intellectual capacities, addressing IPC and AMR within these setting is necessary. Moreover, the majority of the professionals involved in daily care and support for people with ID in the Netherlands do not have a (para)medical background and are trained as social care professionals, even though comorbidities and health problems frequently occur among this group [10]. According to estimates, social care professionals (non-medically trained) account for 52.1% of the total workforce in long-term care for people with disabilities in the Netherlands [22–24].

In the Dutch healthcare system, hospital IPC guidelines are well established, but applying those guidelines in long-term care is often impractical due to a mismatch between the setting and available resources [25, 26]. However, over the last fifteen years, there has been an increased awareness of IPC and antimicrobial stewardship programs to prevent the emergence and spread of AMR. Antimicrobial stewardship programs (AMS) aim to guide healthcare professionals towards optimal selection, dosage, and duration of antimicrobial treatment [27]. Up until now, most studies and surveillance systems on AMR, AMS and IPC focus on LTCFs for older adults [28–31]. Furthermore, the accessibility of training, interventions and resources targeting the long-term care population, with a focus on older adults living in nursing homes or residential care facilities, has significantly improved [32–34]. However, there remains a noticeable lack of attention given to these topics in long-term care settings where people with ID reside.

The current study aims to address this gap by providing insight into the current knowledge, attitudes and perceptions of healthcare professionals working in ID-LTCFs regarding AMR, AMS and IPC. In addition, we aim to explore AMS and IPC practices and arrangements within ID-LTCFs. Gaining insight into the knowledge, attitudes, perceptions, and practices of (medical and nonmedical) healthcare professionals in ID-LTCFs is crucial for optimizing guidelines and implementing effective strategies to control AMR and enhance client safety within this setting.

## Methods

### Study design and setting

We conducted an online cross-sectional survey study including both closed- and open-ended questions to investigate knowledge, attitudes, perceptions and practices regarding AMR, AMS and IPC among healthcare professionals working in ID-LTCFs, in the Netherlands.

### Study sample

In the Netherlands, eight Academic Collaboratives Centers (ACCs) were established by the Dutch Ministery for Health and Welfare to improve health outcomes and quality of life for people with ID through scientific research and enhanced collaboration [35]. ACCs aim to stimulate evidence-based practices and innovation by fostering collaboration between academia and health facilities, people with ID, and professionals working with people with ID. The study sampling started by approaching two ACCs: (1) Academic collaborative Intellectual disability and Health - Sterker op Eigen Benen (SOEB) and (2) Academic collaborative Healthy Ageing in Intellectual Disabilities (GOUD) and asking them to forward survey invitations to organizations within their networks (36, 37). We invited all organizations in the networks of these two ACCs (*N* = 9) to recruit professionals within their network of ID-LTCFs.

### Sampling

The nine care organizations within the networks of the two ACCs actively distributed a weblink for the digital survey environment to healthcare professionals working in affiliated care organizations. We also invited healthcare professionals working in ID care from organizations outside the two ACC networks to participate in this survey. This was done by snowball sampling (by asking the professionals who received the link to the survey to distribute and share with other professionals working in ID-LTCFs within their personal network), LinkedIn posts, the Dutch Association for ID Physicians’ webpage, and the Knowledge Center for Disability Care Netherlands’ webpage. In total 183 care organizations are affiliated with the National Association of Disability Care Netherlands [38].

We aimed to include a representative group of healthcare professionals working in ID-LTCF by collecting responses from at least one, one nurse, and one social care professional per organization, in order to obtain a multiangle overview and perspective from different care specialties. Moreover, (infection control) policy makers and managers were also eligible for enrolment in this study.

### Data collection instrument

The structured web-based survey consisted of a minimum of 74 questions and a maximum of 114 questions (depending on the role of the participant). The survey was made available in Dutch through the survey program Formdesk (https://en.formdesk.com). Digital informed consent was obtained from participants prior to the start of the survey. Participation remained anonymous.

The survey was adapted from validated surveys regarding AMR, AMS and IPC [38–42] and extended with items related to the Dutch ID-care system.

Knowledge survey items regarding AMR, AMS and IPC were based on national guidelines [43]. The survey was tailored to the background and role of the participants. The online survey took 15 to 30 min to complete (depending on the role and background of the participating care professional). Prior to data collection, the study was preregistered using the Open Science Framework (OSF) (10.17605/OSF.IO/AGMFD).

The survey was piloted among 10 healthcare professionals working in ID care (physicians, nurses and social care professionals representative of the target population of this study) and reviewed by several researchers from the National Institute of Public Health and the Environment (RIVM) and Radboud University Medical Center (RadboudUMC).

### Items in the survey

The cross-sectional survey consisted of five parts: (i) information on the objectives of the study and an informed consent page; (ii) sociodemographic details of each participant; (iii) questions regarding AMR, AMS and IPC guidelines and policies; v) statements related to the three KAP domains (knowledge, attitudes and perception) and practices about AMR, AMS and IPC; and vi) willingness to participate in potential follow-up studies.

The KAP survey consisted of 41 statements (knowledge; 10 items, attitudes/experiences; 13 items, perceptions; 10 items and practices; 8 items). The survey items can be found in Supplementary file 1.

### Data analysis

The first step in the data analysis was the assessment of irregularities and missing data prior to the data analysis through visual examination of the dataset.

The overall knowledge scores were assessed using descriptive statistics (n/n, %).

Knowledge sufficiency was assessed by Bloom’s cut-off point, for which we considered knowledge levels to be adequate when more than 80% of the questions were correctly answered [44]. Comparison between medical and nonmedical trained professionals were carried out using the chi-square test.

Items from the attitudes, experiences and perceptions section were scored on a 5-point Likert scale. The means and standard deviations were calculated. Mean scores above three, close to five, indicate agreement with the item surveyed, whereas mean scores below three, and a value closer to one indicated disagreement.

Differences between medical and nonmedical trained professionals were tested using the Mann‒Whitney U test.

Practices regarding hand hygiene, glove use, IPC, AMS, and other (background) variables regarding organizational structures were assessed using descriptive statistics (n/n, %).

The responses to the open-ended questions were coded inductively via thematic analysis. The thematic analysis consisted of open, axial and selective coding [45]. The emerging codes were mapped into categories, resulting into themes (SH). The assigned themes were reviewed by a second researcher and co-author of this study (FH). The coding results were discussed among the two researchers until consensus was reached (SH and FH).

Data analysis was conducted using the Statistical Package for Social Sciences (SPSS) for Windows version 28.0 [46]. The significance level was set at 0.05.

### Ethical considerations

Approval by an ethical research committee or institutional review board was considered unnecessary under current national legislation by the Center for Clinical Expertise at the National Institute for Public Health and the Environment (project number: LCI-620).

The research was conducted in accordance with the principles outlined in the Declaration of Helsinki and the Code of Conduct for Health Research, as well as the General Data Protection Regulation (GDPR).

## Results

A total of 112 participants completed the survey between 13th July and 1st November 2023, 109 of whom were included in the analysis. Three surveys were excluded due to double participation (*n* = 2) or lack of informed consent (*n* = 1).

The characteristics of the participants are presented in Table 1. The participants represented 37 organizations. Based on the total number of organizations represented by the National Association of Disability Care Netherlands (*n* = 183), we have reached approximately 19% of all organizations represented within the National Association of Disability Care Netherlands, including small scale to large care organizations.

**Table 1:** Demographic characteristics of the participants (*n* = 109)

	All participants (*n* = 109)	Medical and paramedical professionals (*n* = 59, 54.1%)	Social care professionals (*n* = 38, 34.9%)	Management and policy professional (*n* = 12, 11.0%)
	n (%)			
**Age**				
18–25 years	6 (5.5%)	3 (5.1%)	3 (7.9%)	0 (0.0%)
26–35 years	22 (20.2%)	11 (18.6%)	9 (23.7%)	2 (16.7%)
36–45 years	33 (30.3%)	17 (28.8%)	12 (31.6%)	4 (33.3%)
46–55 years	30 (27.5%)	20 (33.9%)	9 (23.7%)	1 (8.3%)
56–65 years	18 (16.5%)	8 (13.6%)	5 (13.2%)	5 (41.7%)
Working experience in the current position*				
Less than 5 years	41 (37.6%)	25 (42.2%)	12 (31.6%)	4 (33.3%)
Between 5 and 10 years	19 (17.4%)	11 (18.6%)	6 (15.8%)	2 (16.7%)
Between 10 and 15 years	19 (17.4%)	10 (16.9%)	4 (10.5%)	5 (41.7%)
Over 15 years	29 (26.6%)	13 (22.0%)	15 (39.5%)	1 (8.3%)
**Education**				
Secondary vocational education	33 (30.4%)	10 (17.0%)	22 (57.9%)	1 (8.3%)
Higher professional education	50 (45.9%)	28 (47.5%)	14 (36.8%)	8 (66.7%)
Higher professional and master education	2 (1.8%)	2 (3.4%)	0 (0.0%)	0 (0.0%)
University bachelor (and master education)	23 (20.9%)	19 (32.2%)	1 (2.6%)	3 (25.0%)
Other	1 (0.9%)	0 (0.0%)	1 (2.6%)	0 (0.0%)
**Work setting**				
Working in one fixed location	57 (52.3%)	19 (32.2%)	30 (78.9%)	8 (66.7%)
Working on several locations within one care organization	41 (37.6%)	31 (52.5%)	7 (18.4%)	3 (25.0%)
Working on several locations across different care organizations	11 (10.1%)	9 (15.3%)	1 (2.6%)	1 (8.3%)
**Received education on infection control and prevention**				
Yes	48 (44.0%)	28 (47.5%)	13 (34.2%)	7 (58.3%)
No	58 (53.2%)	30 (50.8%)	23 (60.5%)	5 (41.7%)
I don’t know	3 (2.8%)	1 (1.7%)	2 (5.3%)	0 (0.0%)
**Additional training on antimicrobial resistance**				
Yes	20 (18.3%)	17 (28.8%)	2 (5.3%)	1 (8.3%)
No	88 (80.7%)	42 (71.2%)	35 (92.1%)	11 (91.7%)
I don’t know	1 (0.9%)	0 (0.0%)	1 (2.6%)	0 (0.0%)

*one participant did not indicate working experience in current position, *n* = 108

The number of participants per organization ranged between 1 and 24. Over 50% of the participants were recruited from six organizations. Overall, 54.1% of the participating care professionals were (para)medical professionals (*n* = 18, 16.5% physicians, *n* = 35, 32.1% nurse practitioners, and *n* = 6, 5.5% infection control specialists), 34.9% were social care professionals, and 11.0% were categorized as management or policy professionals (e.g., department managers or health policy makers).

### Knowledge about AMR and IPC

The knowledge levels of most surveyed items, including personal hygiene (e.g., wearing artificial nails, jewellery) and infection transmission, were adequate. However, more than 90% (90/109) of the participants surveyed indicated that they were completely protected against (antibiotic-resistant) bacteria when wearing gloves (**see** Table 2).

**Table 2:** Ten items regarding AMR and IPC based on national guidelines for knowledge assessment (*n* = 109)

	Correct answer	Correctly answered (%)
Wearing artificial nails should be avoided during care moments to prevent the spread of pathogens.	True	96.3
People living in a long-term care facility are more likely to contract a (healthcare-associated) infection than those who do not reside in such a facility.	True	94.5
Wearing jewelry during care moments should be avoided to prevent the spread of pathogens.	True	93.6
Infections caused by a resistant bacterium are no longer treatable at all.	False	89.9
Resistant bacteria (such as MRSA) can be transmitted through hands.	True	89.0
Bacteria primarily spread through the air.	False	87.2
A damaged skin should be covered during care moments (e.g., with a bandage) to prevent the spread of pathogens.	True	84.4
Clients carrying a (antibiotic-resistant) bacterium (such as MRSA) are always treated for it.	False	74.3
Regular use of hand cream during care moments should be avoided to prevent the spread of pathogens.	False	72.5
Wearing gloves provides me with complete protection against (antibiotic-resistant) bacteria.	False	9.2

Overall, 65.1% of the participants exhibited sufficient knowledge of AMR and IPC based on Bloom’s cut-off. The percentage of participants with sufficient overall knowledge was significantly greater among (para) medical professionals (74.6%) than among social care professionals (52.6%) (*p* = 0.026), see Supplementary file 2 for the percentages split per job role.

### Experiences and attitudes about AMR and IPC

High mean scores were observed for items regarding IPC protocols (4.2), hand hygiene (4.3 and 4.4) and the feeling of responsibility towards clients to prevent the transmission of bacteria (4.5). However, low mean scores were observed for items related to perceived accountability from colleagues for hand and personal hygiene (2.4), as well as beliefs that supervisors and management can do more to promote IPC among employees within the organization (2.6 and 2.7). There was a statistically significant difference between the (para)medical professionals and social care professionals regarding their *self-indicated ability to adhere to IPC protocols* (mean scores: 4.0 versus 3.6, p value: *0.03*) and their *belief that IPC receives enough attention within the organization* (mean scores: 3.0 versus 2.6, p value: *0.03*). Table 3.

**Table 3:** Experiences and attitudes regarding AMR and IPC measured on a 5-point likert scale and presented as the mean and standard deviation

	Overall Mean (SD)	(Para)medical professional Mean (SD)	Social professional Mean (SD)	Management and policy professional Mean (SD)	Difference based on subgroup: (para)medical vs. social professional *P* value (Test statistic)
I know where to find our work protocols when I have doubts about infection prevention and control.	4.2 (0.9)	4.3 (0.8)	4.0 (0.9)	4.7 (0.5)	0.12 (1.57)
I am able to follow the advice from infection prevention and control protocols effectively.	3.8 (0.8)	4.0 (0.7)	3.6 (0.8)	4.0 (0.9)	0.03* (2.18)
I feel responsible for preventing the transmission of bacteria to other clients.	4.5 (0.6)	4.5 (0.7)	4.5 (0.6)	4.5 (0.5)	0.73 (0.34)
There are enough resources and materials to execute the infection prevention and control protocols.	3.8 (1.0)	3.9 (1.0)	3.7 (0.9)	4.2 (1.1)	0.31 (1.02)
I practice hand hygiene in my work.	4.4 (0.7)	4.4 (0.8)	4.3 (0.7)	4.5 (0.7)	0.22 (1.24)
It is clear to me when I should and should not apply hand hygiene.	4.3 (0.7)	4.4 (0.7)	4.1 (0.9)	4.6 (0.7)	0.08 (1.74)
I bundle (care) tasks to reduce the frequency of hand hygiene.	3.0 (1.1)	2.9 (1.1)	2.8 (1.0)	4.3 (0.8)	0.75 (0.32)
My colleagues hold me accountable when I do not adhere to hand hygiene and personal hygiene.	2.4 (1.1)	2.5 (1.1)	2.4 (1.2)	2.5 (1.2)	0.90 (0.30)
I address my colleagues when I notice they are not following hand hygiene and personal hygiene measures.	3.4 (1.1)	3.5 (1.0)	3.2 (1.1)	3.8 (1.2)	0.27 (1.10)
I follow the guideline regarding personal hygiene in my work.	4.0 (1.0)	4.1 (0.9)	3.8 (1.0)	4.4 (0.7)	0.15 (1.45)
I believe that infection prevention and control receives enough attention in my organization.	2.9 (1.0)	3.0 (0.1)	2.6 (1.0)	3.2 (0.8)	0.03* (2.20)
I believe that supervisors do enough to promote infection prevention and control among employees on the floor.	2.6 (1.0)	2.6 (1.1)	2.5 (1.0)	2.8 (1.0)	0.51 (0.66)
I believe that the management does enough to promote infection prevention and control among employees on the floor.	2.7 (1.0)	2.7 (1.0)	2.5 (1.0)	3.1 (1.2)	0.36 (0.92)
Mean all items	3.4 (0.7)	3.5 (0.7)	3.3 (0.6)	3.2 (0.9)	0.12 (1.56)

The answer options on the 5-point Likert scale were coded as follows: [5; strongly agree, 4; agree, 3; neither agree nor disagree, 2; disagree, 1; strongly disagree]. Participants who answered “not applicable n/a’’ are excluded from the mean and standard deviation calculations. The mean score can be interpreted as such. *Statistically significant difference between groups found at the p level of 0.05. *The test statistic used to compare subgroups was the Mann‒Whitney U test, which tested whether the distribution of the individual tested item was the same across the two subgroups (medical vs. social professionals) (null hypothesis)*. The significance level was set at *P< -0.05.*

### Perceptions of AMR and IPC

High mean scores were observed for items regarding personal hygiene (4.1), the effectiveness of IPC to protect clients (4.1), the necessity of IPC to protect clients (4.2) and hand hygiene (4.5). Moreover, participants disagreed with the statement that hand and personal hygiene potentially impede the sense of homeliness (home-like setting) for people with ID (2.2 and 2.4). There was a statistically significant difference between the (para)medical professionals and social care professionals regarding the *overall mean score for all perception items* (3.5 versus 3.3, p value: 0.02), *organizations of IPC* (mean scores: 4.0 versus 3.6, p value: 0.02), *importance of personal hygiene* (mean scores: 4.3 versus 3.7, p value: 0.00), *whether personal hygiene guidelines hinder homeliness* (man scores: 2.2 versus 2.7, p value: 0.03) and *whether clients are at high risk of acquiring AMR bacteria during care* (mean scores: 3.1 versus 2.6, p value: 0.00). (see Table 4**).**

**Table 4:** Mean scores on perceptions of AMR and IPC as measured on a 5-point likert scale

	Overall Mean (SD)	(Para)medical Mean (SD)	Social professional Mean (SD)	Management and policy professional Mean (SD)	Difference based on subgroup: (para)medical vs. social professional *P* value (Test statistic)
I think antibiotic resistance is a significant problem	3.9 (0.9)	4.0 (0.9)	3.9 (0.8)	4.1 (0.8)	0.33 (0.97)
I think infection prevention and control measures are necessary to prevent the spread of (resistant) bacteria	4.2 (0.7)	4.2 (0.7)	4.0 (0.7)	4.3 (0.8)	0.11 (1.61)
I think infection prevention and control measures protect the client from getting an infection.	4.1 (0.7)	4.1 (0.8)	3.9 (0.7)	4.3 (0.8)	0.20 (1.26)
I think good organization of infection prevention and control within an institution prevents the spread of pathogens (bacteria).	3.9 (0.8)	4.0 (0.8)	3.6 (0.8)	4.1 (0.9)	0.02* (2.28)
I consider hand hygiene important in my work.	4.5 (0.6)	4.6 (0.6)	4.3 (0.6)	4.5 (0.8)	0.08 (1.73)
I believe that hand hygiene hinders homeliness.	2.2 (0.9)	2.0 (0.9)	2.4 (1.0)	2.4 (1.1)	0.09 (-1.70)
I consider personal hygiene important in my work (removing jewelry, tying back hair, possibly wearing work clothing)	4.1 (0.9)	4.3 (0.7)	3.7 (1.0)	4.2 (0.9)	0.00* (2.98)
I believe that personal hygiene guidelines hinder homeliness.	2.4 (1.0)	2.2 (0.9)	2.7 (1.1)	2.7 (1.3)	0.03* (-2.21)
I believe I am at high risk of acquiring a (resistant) bacterium during my work.	2.5 (0.9)	2.6 (0.8)	2.3 (1.0)	2.7 (1.0)	0.08 (1.74)
I believe my clients are at high risk of acquiring a (resistant) bacterium during care.	3.0 (0.9)	3.1 (0.9)	2.6 (1.0)	2.9 (0.8)	0.00* (2.84)
Mean all items	3.4 (0.4)	3.5 (0.3)	3.3 (0.4)	3.6 (0.4)	0.02* (2.39)

The answer options on the 5-point Likert scale were coded as follows: [5; strongly agree, 4; agree, 3; neither agree nor disagree, 2; disagree, 1; strongly disagree]. Participants who answered “not applicable n/a’’ are excluded from the mean and standard deviation calculations. The mean score can be interpreted as such. *Statistically significant difference between groups at the p level of 0.05.

### Self-reported hand hygiene practices

**Fig. 1:**
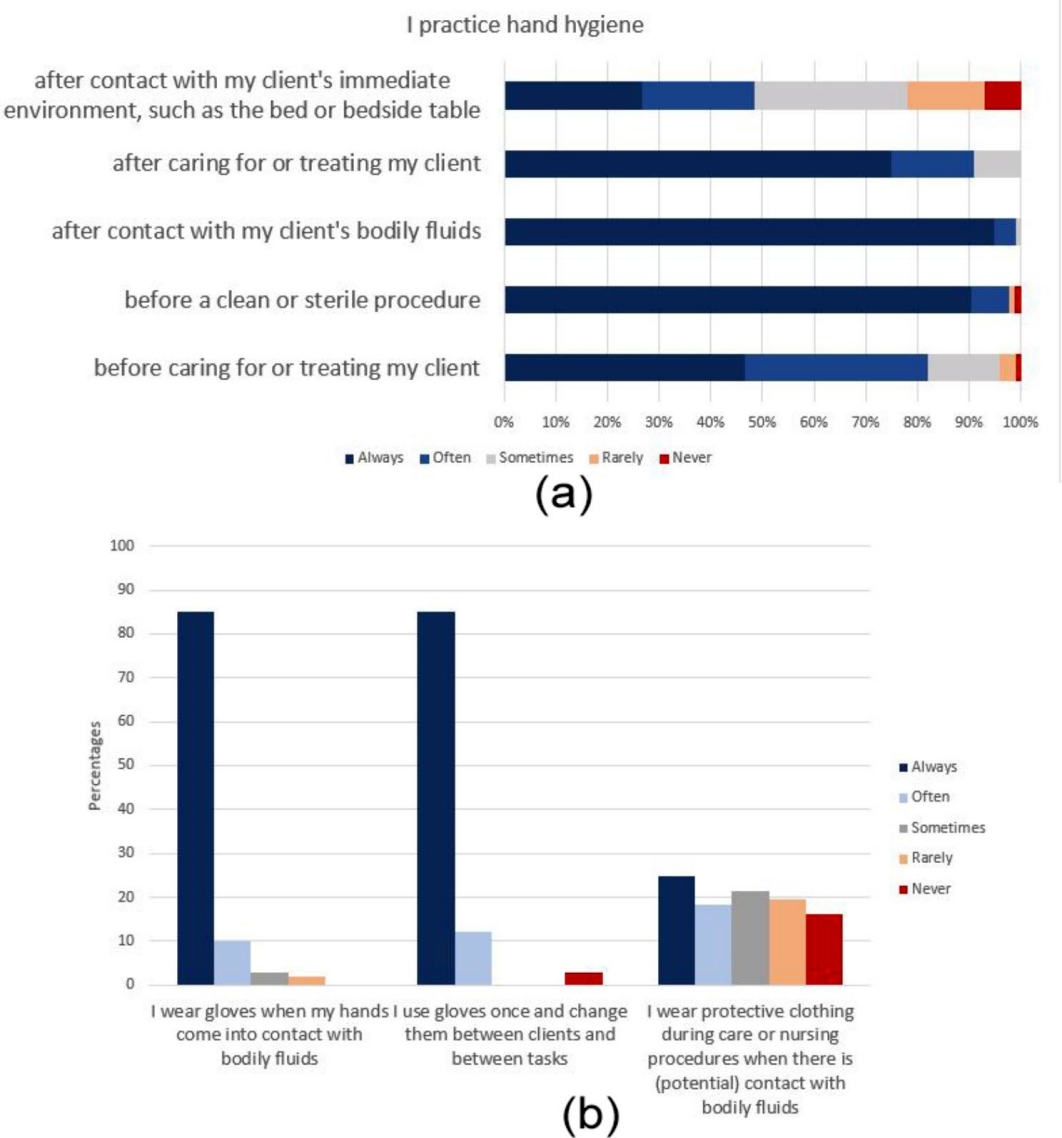
**a**. Hand hygiene at five time points was assessed based on survey items. **b**. Three items assessing self-reported practices regarding glove use and personal protective equipment (PPE) use

Self-reported hand hygiene compliance after contact with the client’s immediate environment was low, with only 48.5% of participants indicating consistent adherence in this context (Fig. 1a).

### Self-reported glove use and personal protective equipment (PPE) use

Only 43% of participants reported consistent use of PPE, specifically during care or nursing procedures involving potential contact with bodily fluids (PPE use is necessary in this situation according to the Dutch IPC guidelines) (Fig. 1b). Social professionals reported the lowest PPE usage at 20.0%, followed by (para)medicals at 56.9% and management and policy professionals at 57.1%.

### Awareness, guidelines and organizational structures of AMR and IPC

The survey results highlight nurses as the primary group responsible for IPC. When an infection prevention and control committee was available, this committee was generally composed of physicians, nurses, policy and quality officers, and healthcare managers. Notably, infection prevention specialists are often not represented within infection prevention and control committees across the organizations surveyed.

One-third of the participants (28/85, 32.9%) believed that current national infection control and hygiene guidelines were not easily applicable to the sector.

Participants mentioned that the existing guidelines were commonly adapted from the hospital setting and do not align with the personalized, setting-specific needs of ID-LTCFs.

In addition, most protocols were not easy for clients (those with ID). This was experienced as a challenge to instruct clients and inform them about infection control and hygiene guidelines.

The majority of the participants (> 90%) thought that AMR and IPC need more attention within the disability care sector, but paradoxically, only 38.5% mentioned that they would like to receive additional information and training about IPC, and 72.5% would like to receive additional information and training about AMR.

Findings from open field questions showed that participants believed that antibiotics are often prescribed for ambiguous symptoms, which potentially leads to unnecessary and excessive use of antimicrobial agents. Moreover, they described that improved expertise in this area would greatly contribute to comprehending infectious diseases or outbreaks that require early monitoring and response.

### Antimicrobial prescribing and stewardship (AMS)

Thirty-five individuals were surveyed regarding antibiotic prescription practices, with 16 out of 35 participants (45.7%) reporting prescribing antimicrobial agents within their role. Two-thirds (10/16, 62.5%) exclusively follow the Dutch College of General Practitioners (NHG) guidelines for the diagnosis and treatment of infections, and one-third uses local and/or other resources, sometimes combined with NHG guidelines.

Based on findings from the open field questions, one-third of participants reported that prescribing guidelines were not suitable for ID-LTCFs, given the complexity of care, challenges in obtaining biological samples such as blood or urine, polypharmacy or known resistance, and the fact that a substantial proportion of people with disabilities suffer from dysphagia.

Fourteen out of sixteen participants consulted a medical microbiologist, and fifteen out of sixteen participants mentioned that they contacted the pharmacists to primarily discuss pharmaceutical interactions.

## Discussion

In this cross-sectional survey we found that healthcare professionals working in ID-LTCFs acknowledge the importance of AMR and IPC. Nevertheless, there is room for improvement, particularly in terms of the perceived protective value of gloves against microorganisms and the translation of guidelines into easily comprehensible and practical information for both healthcare professionals and individuals with ID. This is imperative to address as previously reported findings in other long-term care settings are not generalizable to the Dutch long-term care setting for people with ID due the unique nature of the setting and demographics of the individuals living in this setting.

The results of this study illustrate that while most participants were aware of basic infection control and hygiene measures, only two-thirds showed adequate knowledge levels, which is in line with findings from other healthcare settings [46–49]. When comparing (para)medical and social care professionals, we discovered significant differences in AMR, AMS and IPC knowledge, attitudes and perceptions. This finding was not surprising as social care professionals in the Netherlands have a nonmedical background. Moreover, we found remarkable results regarding perceptions of glove use: over 90% of the participants believed that wearing gloves completely protected them from acquiring or spreading antimicrobial-resistant bacteria. Gloves serve as a barrier to prevent direct contact with microorganisms, such as AMR bacteria. However, glove utilization does not guarantee that any contact while wearing gloves prevents the spread of bacteria. Incorrect glove use, removal or disposal might result in cross-contamination, decreasing its protective value [50]. This indicates a gap in participants’ understanding of the limitations of this protective measure and emphasizes the need for more awareness about correct glove use. To our knowledge, this is the first study reporting this finding in the ID-LTCF setting. Furthermore, self-reported use of PPE was found to be relatively low. This finding aligns with previous observational and self-reported survey studies in long-term care settings, which also highlighted suboptimal compliance with PPE [47, 49–52].

Regarding IPC training in ID-LTCFs, we reported that 47.5% of the medical and paramedical professionals received IPC training, and based on findings from the European HALT study, 79% of Irish nursing and paramedical personnel working in Irish ID-LTCFs reported having received IPC training [53]. As the HALT study is the only study a in literature reporting on IPC training in ID-LTCFs, it is worth mentioning. However, it is unclear whether the group of participants is comparable to the professionals included in our study, which hinders direct comparisons between the Irish HALT survey from 2013 and our study. In the Netherlands, samples of long-term care facilities for older adults have participated in the HALT survey over the last years [31]. However, until now, ID-LTCFs were not recruited in the Netherlands. Given the insights from this study, including ID-LTCFs into future HALT surveys must be considered. In this context, the results of the current study show it remains important for HALT to report the results of LTCFs for the elderly and ID-LTCFs separately, allowing the sectors to use their specific findings to improve infection prevention policies.

The participants in our study highlighted the necessity for clearer guidelines tailored to the ID-LTCF setting and easily understandable information for individuals with disabilities. Such materials are currently lacking within the existing IPC and hygiene policies. Easy-to-read materials are, however, critical for people with disabilities because they facilitate awareness, empower autonomy, and ensure that information about hygiene practices and IPC guidelines is accessible and understandable. Hence, developing and disseminating such materials may allow them to participate more actively in preventive healthcare [54, 55]. Greater efforts are needed to ensure inclusive IPC and hygiene materials for people with ID.

In the ID-LTCFs surveyed, frequent exchanges of healthcare professionals across different locations were indicated. This practice of working in multiple locations has the potential to negatively impact adherence to IPC, AMR and AMS procedures by influencing the consistency and standardization of policies across many care locations and settings, departments, and work environments [56]. Simultaneously, one could argue that working in different places is beneficial to the quality of care since best practices can be identified and put into practice elsewhere. On the contrary, competing organizational priorities may divert attention and resources away from IPC, AMS and AMR, resulting in less attention and organizational priority towards these subjects. The varying emphasis on roles and institutional hierarchies can also influence how different healthcare professionals see, support and comply with IPC and AMS interventions [57]. While many facilities had numerous policies and procedures in place for IPC, there appeared to be a notable absence of the structures and leadership required for their sustained and effective long-term implementation, which has also been addressed by other authors researching IPC in the LTCF setting [40, 57–61].

While many of the organizations in our study established infection control committees, infection prevention specialists were not always represented within these committees. The importance of these experts’ involvement should be emphasized more, and a proactive approach that puts them at the forefront of prevention is vital, rather than limiting their involvement during outbreaks or active cases [62]. These infection prevention specialist can also assist during the development and/or implementation of local IPC guidelines [63].

As residents of LTCFs often share communal living areas and sanitary facilities (which can be a potential reservoir for microorganisms), complying with IPC guidelines becomes critical in minimizing pathogen spread within these facilities [30, 64]. The challenge in developing IPC and AMS guidelines that can be applied to and implemented in ID-LTCFs is made more difficult by the considerable heterogeneity among this group. Factors contributing to this heterogeneity include: “i) differences in terms of organizational structures within long-term care facilities or organizations; ii) different levels of assistance and nursing intensity (for example, complex medical care versus assisted living), iii) the size of LTCFs, and iii) access to physicians’ input and diagnostic testing”, as previously reported by Smitt et al. [25]. This heterogeneity underlines the importance for tailored interventions and practices.

In addition to heterogeneity, the home-like environment may hinder compliance with IPC and hygiene measures in ID-LTCFs. It is not only about preventing or controlling infection but also about developing policies that prioritize the well-being of residents. Navigating this terrain involves a careful balance between infectious disease management, risk for potential transmission and the overall well-being of people with ID [64–68].

The strengths of our study includes (i) the use of validated items in the survey, (ii) a diverse sample of participants from various ID-LTCFs, (iii) information about participants’ interest in future IPC and AMR research in ID-LTCFs, allowing for possible follow-ups beyond and within this sample, and (iv) the use of both closed- and open-ended questions, which revealed new areas for further research, such as client engagement. However, the findings of our study may be limited by (i) social desirability bias due to the nature of self-administered digital surveys, potentially leading to an overestimation of positive reported attitudes and practices; (ii) selection bias, as some participants are interested and familiar with this topic, possibly leading to findings that may not be generalizable to all healthcare professionals working in ID-LTCFs and selection bias introduced by recruitment strategy, as half of the participants were included from six of the 37 organizations and 24 responses were included from one participating organization. This may limit representativeness of the study findings. However, these 24 participants were included from the largest organization providing care to people with disabilities in the Netherlands and roughly 19% of all care organizations represented by the The National Association of Disability Care Netherlands are included in our study sample ; (iii) self-report survey design, which limits the accurate assessment of practices and introduces some uncertainties around self-reported compliance; and (iv) the Dutch healthcare context, which is solely studied. Still, the study design and our measurement instrument may be useful for other settings and countries to study AMR, AMS and IPC.

To further elaborate on these insights, future research should focus on the underlying factors that influence the knowledge, attitudes, perceptions and practices of healthcare professionals working in ID-LTCFs, particularly healthcare professionals with nonmedical backgrounds, such as social care professionals (the largest professional group working in Dutch ID-LTCFs), medical professionals, and, ideally, people with ID. Moreover, it is essential to engage with environmental service workers such as cleaners or maintenance workers as hygiene plays a crucial role in disrupting AMR transmission within the long-term facility. This can be achieved by providing an in-depth understanding of the perceived barriers and facilitators of AMR, AMS and IPC among these different groups. Moreover, it would be interesting to assess how healthcare professionals and people with ID deal with AMR, AMS and IPC in practice, by routine audit of IPC behaviors to assess compliance, and how they comply to IPC guidelines for example, by conducting prospective observational studies.

In the coming years, tailored IPC guidelines will be developed for the Dutch ID-care setting in close collaboration with the Dutch Association for ID-physicians [69]. Findings from this and future studies may inform policymakers about targeted educational strategies and guidelines, considering inclusivity, and addressing the unique needs of this population, potentially leading to setting-specific policies and, ultimately, improved health outcomes for people with disabilities.

## Conclusion

Healthcare professionals working in ID-LTCFs acknowledge the importance of IPC in preventing disease and containing the emergence and spread of AMR. Continued efforts are needed to make IPC guidelines more accessible to all healthcare professionals working in ID-LTCFs and people with ID themselves. Future studies and interventions should focus on appropriate glove use and setting-specific IPC and hygiene arrangements and policies. It is important to continuously assess the needs of healthcare professionals, people with disabilities, and other key stakeholders, as these insights have great potential for the development and implementation of novel and inclusive AMR, AMS and IPC guidelines and policies.

### Electronic supplementary material

Below is the link to the electronic supplementary material.

Supplementary Material 1

Supplementary Material 2

## Data Availability

The data collected and analysed during the current study are available from the corresponding author on reasonable request.
