# Supplementary material for: Mind the gap: knowledge, attitudes and perceptions on antimicrobial resistance, antimicrobial stewardship and infection prevention and control in long-term care facilities for people with disabilities in the Netherlands
Source: Antimicrob Resist Infect Control. 2024 Jun 5;13:56. doi: 10.1186/s13756-024-01415-3 (PMC11151466; doi:10.1186/s13756-024-01415-3)
Supplement: Supplementary file 1 — Supplementary Material 1 [file 13756_2024_1415_MOESM1_ESM.docx]

**Survey instrument: Infection prevention and antimicrobial resistance in long-term care for people with disabilities**

Questions in black were displayed to all respondents.
Questions in brown were only displayed to physicians and managers.

Demographic characteristics of respondent (displayed to ALL job roles)

| What is your age? | [..] |
| --- | --- |
| What is your highest level of education? | - Middle level applied education 2 - Middle level applied education 3 - Middle level applied education 4 - Higher education: bachelor - University: bachelor - University: bachelor and master - In-house training - Other: [..] |
| What is your current position? | - Ambulatory Support Worker - Social worker - Senior social worker - Pedagogical Assistant - Social Pedagogical Counselor - Assistant Nurse - Caregiver - Nurse - Advanced Practice Nurse - Quality Assurance Specialist/Prevention Specialist - Infection Prevention Advisor/ - Infection Prevention Expert - Intellectual Disability Doctor (ID Doctor) - Trainee Intellectual Disability Doctor (ID Doctor) - General Practitioner (GP) - Junior Doctor - Team Leader/Supervisor - (Healthcare) Manager |
| How many years are you working in your position? | [..] |
| Do you work in one organization or multiple? | - I work in one organization, on one location - I work in multiple organizations, on one location - I work in multiple organizations, on multiple locations |
| In which care organization are you currently employed? | - s Hereen Loo - Koraal - Dichterbij - Driestroom - Philadelphia - Siza - Abrona - Amarant - Ipse de Bruggen - Other [..] |
| Which clients group do you work with? Check all options that apply to your situation | - Clients with a mild intellectual disability  - Clients with a moderate to severe intellectual disability  - Clients with a severe multiple disability  - Clients with acquired brain injury (ABI)  - Clients with a visual impairment  - Clients with autism |
| How many residents are within your organization with moderate to severe multiple disabilities? | - 0-20 - 20-50 - 50-80 - More than 80 |
| How many residents are within your organization overall? | - 0-100 - 100-500 - 500-1000 - Over 1000 |
| Is there a medical services department within your organization? | - Yes - No - I don’t know |
| If yes, which specialties are represented in your medical services department? | - Physician (ID) - General practioners - Junior doctor - Nurse (specialized ID) - Nurse general - Nurse assistant - Other [..] |

Infection prevention and antimicrobial resistance: organization and education (displayed to ALL job roles)

Introduction: The following questions pertain to the organization and training related to infection prevention and antibiotic resistance. When we refer to infection prevention, we mean, for example, hand hygiene, personal hygiene, and the use of personal protective equipment.

| Was infection prevention a topic that received attention during your previous education? | - Yes - No - I don’t know |
| --- | --- |
| Was antimicrobial resistance a topic that received attention during your previous education? | - Yes - No - I don’t know |
| Did you receive any training about infection prevention in your current job? | - Yes - No - I don’t know |
| If yes, when was this training? | - Less than 12 months ago - Between 12 and 24 months ago - Between 24 and 36 months ago - More than 36 months ago |
| If yes, could you elaborate more on this training, what was it about? | [..] |
| If yes, how did you like the training? | [..] |
| Did you receive any training about antimicrobial resistance in your current job? | - Yes - No - I don’t know |
| If yes, When was this training? | - Less than 12 months ago - Between 12 and 24 months ago - Between 24 and 36 months ago - More than 36 months ago |
| If yes, Could you elaborate more on this training, what was it about? | - [..] |
| If yes, How did you like the training? | - [..] |
| Is there anyone responsible for infection prevention policy within your organization? | - Yes - No - I don’t know |
| If yes, Which role does this person have within the organization? | [..] |
| If no, Why do you think that no one is responsible for infection prevention in your organization? | [..] |

Infection prevention and control committee (displayed to ALL job roles)

| Is there an infection prevention and control committee available within your organization? | - Yes - No - I don’t know |
| --- | --- |
| If yes, who is part of this committee. Which specialties are represented? | [..] |
| If yes, is this a fixed committee? | - Yes - No - I don’t know |
| Does this committee regularly come together? |  |
| Is there an infection prevention advisor within your organization who’s responsibility is to guide infection prevention and control? | - Yes - No - I don’t know - Other [..] |
| If yes, which background does this colleague have? | - Nurse assistant - Nurse - Social worker - Physcian |

Infection prevention/hygiene guidelines and work protocols (displayed to ALL job roles)

Introduction: The following questions will be about the presence and use of infection prevention/hygiene guidelines within your organization

| Do you use infection prevention guidelines in your daily work? | - Yes - No |
| --- | --- |
| Which infection prevention guidelines do you use? | - RIVM - NHG - V&VN - VerenSO - Other: [..] |
| Are the guidelines available in short, easy to read instructions within your organization? | - Yes - No - I don’t know |
| If yes, how many times do you consult these instructions/protocols? | - Never - Sometimes - Frequently - All the time |
| For which of the following subjects are protocols available within your organization? | - Hand hygiene - Personal Hygiene - Use of personal protective equipment - Multi resistant *Staphylococcus aureus* - Multi-drug resistant organisms - I don’t know - Not applicable |
| Do you think that these protocols are suitable and applicable to the long-term care setting for people with (intellectual) disabillities? | - Yes - No |
| Please elaborate on your answer | [...] |
| Do you believe that infection prevention and hygiene within intellectual disability care deserve more attention? | - Yes - No |
| Please elaborate on your answer | - [...] |
| Do you yourself need more information about infection prevention and hygiene? |  |
| Please elaborate on your answer | - [...] |

Multi-resistant *Staphylococcus aureus* (MRSA) (displayed to medical professionals only)

| Are you familiar with the WIP (Infection Prevention Working Group) MRSA guideline, MRSA manual, and/or MRSA action plan from the RIVM (National Institute for Public Health and the Environment)? | - Yes - No |
| --- | --- |
| Have you ever used the MRSA guideline, manual, or action plan from the RIVM? | - Yes - No |
| How often have you come into contact with resistant bacteria in your work, such as MRSA? | - Never - Occasionally (1-3 times) - Frequently (more than 3 times) - I don’t know |

Antibiotic resistance (displayed to ALL job roles)

| Do you believe that antimicrobial resistance within intellectual disability care deserves more attention | - Yes - No |
| --- | --- |
| Please elaborate on your answer | - [...] |
| Do you yourself need more information about antimicrobial resistance? |  |
| Please elaborate on your answer | - [...] |

Antimicrobial prescribing (displayed to medical professionals only)

Introduction. Are you a treating physician, such as a physician for individuals with intellectual disabilities, a general practitioner, or a nurse specialist? If so, we are interested in your experiences with prescribing antibiotics, particularly for the client group with severe (multiple) intellectual disabillities

| Do you ever prescribe antibiotics within your organization to your clients? | - Yes (only when answered yes, the participants moves to the next question) - No |
| --- | --- |
| If yes, which guideline or formulary do you use when prescribing antibiotics? | - NHG guideline - VerenSo guideline - Formulary (e.g., Groningen 2019) - Other, please specify: [..] |
| Does the guideline or formulary you use align with the treatment plan of your clients? | - Yes - No |
| Please provide your reasoning. | [..] |
| In case of a suspected lower respiratory tract infection I […] prescribe antibiotics  In case of a suspected urinary tract infection I […] prescribe antibiotics | - Always - Mostly - Sometimes - Some cases - Never |
| In case of a suspected skin infection I […] prescribe antibiotics | - Always - Mostly - Sometimes - Some cases - Never |

Antimicrobial stewardship (AMS) (displayed to medical professionals only)

| Do you ever have contact with a clinical microbiologist regarding your patient's treatment plan? | - Yes - No |
| --- | --- |
| If yes, can you provide an example of a contact moment with the clinical microbiologist? | [..] |
| If no, would you have a need for advice from a clinical microbiologist regarding your patient's treatment plan (e.g., on which antibiotic would be best to use for your patient's treatment) | - Yes - No - Not applicable |
| Do you ever have contact with a pharmacist regarding your patient's treatment plan? | - Yes - No |
| If yes, can you provide an example of a contact moment with the pharmacist? | [..] |
| If no, would you have a need for advice from a pharmacist regarding your patient's treatment plan (e.g., on combination of medication and treatment) | - Yes - No - Not applicable |
| Where would you like to learn more? You can check multiple answers." | - Diagnostics of infections in clients with ID (Intellectual Disabilities) - (Proper) antibiotic use - Antibiotic resistance |

Knowledge about infection prevention control and antimicrobial resistance (displayed to ALL job roles)

Introduction. In this section, statements are presented to you, and you can answer with 'true' or 'false'. The statements are about your knowledge of infection prevention and antibiotic resistance. You can only fill in one option at a time, and your initial instinct is the best.

| Which of the following statements are true? | True | False |
| --- | --- | --- |
| Wearing jewelry during care moments should be avoided to prevent the spread of pathogens.  A damaged skin should be covered during care moments (e.g., with a bandage) to prevent the spread of pathogens.  Wearing artificial nails should be avoided during care moments to prevent the spread of pathogens.  Regular use of hand cream during care moments should be avoided to prevent the spread of pathogens.  Wearing gloves provides me with complete protection against (antibiotic-resistant) bacteria.  Infections caused by a resistant bacterium are no longer treatable at all.  Clients carrying a (antibiotic-resistant) bacterium (such as MRSA) are always treated for it.  Bacteria primarily spread through the air.  Resistant bacteria (such as MRSA) can be transmitted through hands.  People living in a long-term care facility are more likely to contract a (healthcare-associated) infection than those who do not reside in such a facility. |  |  |

Attitudes, experiences and practices (displayed to ALL job roles)

Introduction. In this section, statements are presented to you, and you can indicate on a five point Likert scale from 'completely disagree-disagree-neutral-agree-completely agree or not applicable’ what you think of the statement. The first part of these statements is about **your experiences in daily practice** with infection prevention and antibiotic resistance. The second part is about **your perceptions**. Your initial instinct is the best.

Part 1: Experiences in practice

| I know where to find our work protocols when I have doubts about infection prevention.  I am able to follow the advice from infection prevention protocols effectively.  I feel responsible for preventing the transmission of bacteria to other clients.  There are enough resources and materials to execute the infection prevention protocols.  I practice hand hygiene in my work.  It is clear to me when I should and should not apply hand hygiene.  I bundle (care) tasks to reduce the frequency of hand hygiene.  My colleagues hold me accountable when I do not adhere to hand hygiene and personal hygiene.  I address my colleagues when I notice they are not following hand hygiene and personal hygiene measures.  I follow the guideline regarding personal hygiene in my work.  I believe that infection prevention receives enough attention in my organization.  I believe that supervisors do enough to promote infection prevention among employees on the floor.  I believe that the management does enough to promote infection prevention among employees on the floor. |
| --- |

Perception

| I think antibiotic resistance is a significant problem.  I think infection prevention measures are necessary to prevent the spread of (resistant) bacteria.  I think infection prevention measures protect the client from getting an infection.  I think good organization of infection prevention within an institution prevents the spread of pathogens (bacteria).  I consider hand hygiene important in my work.  I believe that hand hygiene hinders homeliness.  I consider personal hygiene important in my work (removing jewelry, tying back hair, possibly wearing work clothing).  I believe that personal hygiene guidelines hinder homeliness.  I believe I am at high risk of acquiring a (resistant) bacterium during my work.  I believe my clients are at high risk of acquiring a (resistant) bacterium during care. |
| --- |

Hand hygiene in practice

Introduction. Statements about hand hygiene in practice are presented to you, and you can indicate on a scale from 'never' to 'always' what you think of the statement. These statements are about hand hygiene and the use of personal protective equipment.

|  | Never, Rarely, Sometimes, Often, Always, N/A |
| --- | --- |
| I practice hand hygiene ...  ... before caring for or treating my client.  ... before a clean or sterile procedure.  ... after contact with my client's bodily fluids.  ... after caring for or treating my client.  ... after contact with my client's immediate environment, such as the bed or bedside table. |  |
| I wear gloves when my hands come into contact with bodily fluids. |  |
| I use gloves once and change them between clients and between tasks. |  |
| I wear protective clothing during care or nursing procedures when there is (potential) contact with bodily fluids. |  |

General questions (displayed to ALL job roles)

You have reached the end of the questionnaire, and to conclude, we have a few general questions.

Which topics within long-term care for individuals with disabilities do you believe require more attention?

Please rank the following topics (1 being the most important and 5 being the least important):

You can choose from the following topics:

- Hand hygiene
- Personal hygiene
- Personal protective equipment
- (Proper) use of antibiotic
- Resistance information (e.g., location and facility level)

Are you familiar with ABR (antibiotic resistance) care networks in your region (e.g., GAIN region Gelderland, LINK region Limburg)?

- Yes
- No

Do you have an interest in one or more of the activities? Please check which activities we may contact you for in the future. You can select more than one activity:

| Insight into your own (local) antibiotic prescription data compared to national benchmarks using pharmacy dispensing information. |
| --- |
| Insight into your own (local) resistance data using laboratory information. |
| Participation in an interview about antibiotic prescription. |
| Participation in an interview or focus group discussion about infection prevention in long-term care for individuals with intellectual disabilities. |
| Participation in a biennial advisory group where researchers will seek your experiences and expertise. |
| I have no interest in the above activities and do not wish to be contacted in the future. |

Would you like to stay informed about future research and research results through a newsletter?

- Yes, if yes please provide us with your e-mail credentials
- No

"Thank you very much for your cooperation; your input is highly valuable! If you have any comments about this questionnaire, you can provide them here."

- **END -**
