# Supplementary material for: Mind the gap: knowledge, attitudes and perceptions on antimicrobial resistance, antimicrobial stewardship and infection prevention and control in long-term care facilities for people with disabilities in the Netherlands
Source: Antimicrob Resist Infect Control. 2024 Jun 5;13:56. doi: 10.1186/s13756-024-01415-3 (PMC11151466; doi:10.1186/s13756-024-01415-3)
Supplement: Supplementary file 2 — Supplementary Material 2 [file 13756_2024_1415_MOESM2_ESM.docx]

**Findings from knowledge section split by occupation type (role within organization)**

- **Medical** represents medically trained professionals
- **Socia**l represents social care professionals
- **Others** represents management and policy professionals
